# Rapid Identification of the Geographical Origin of the Chinese Mitten Crab (*Eriocheir sinensis*) Using Near-Infrared Spectroscopy

**DOI:** 10.3390/foods13203226

**Published:** 2024-10-10

**Authors:** Renhao Liu, Qingxu Li, Hongzhou Zhang

**Affiliations:** 1 College of Mechanical and Electrical Engineering, Tarim University, Alar 843300, China; k4su59@163.com; 2 College of Computer Science, Anhui University of Finance & Economics, Bengbu 233030, China; 120220059@aufe.edu.cn

**Keywords:** Chinese mitten crab, geographical origin, near-infrared spectroscopy, rapid detection

## Abstract

The Chinese mitten crab (*Eriocheir sinensis*) is highly valued by consumers for its delicious taste and high nutritional content, including proteins and trace elements, giving it significant economic value. However, variations in taste and nutritional value among crabs from different regions lead to considerable price differences, fueling the prevalence of counterfeit crabs in the market. Currently, there are no rapid detection methods to verify the origin of Chinese mitten crabs, making it crucial to develop fast and accurate detection techniques to protect consumer rights. This study focused on Chinese mitten crabs from different regions, specifically Hongze Lake, Tuo Lake, and Weishan Lake, by collecting near-infrared (NIR) diffuse reflectance spectral data from both the abdomen and carapace regions of the crabs. To eliminate noise from the spectral data, pretreatment was performed using Savitzky–Golay (SG) smoothing, Standard Normal Variate (SNV) transformation, and Multiplicative Scatter Correction (MSC). Key wavelengths reflecting the origin of Chinese mitten crabs were selected using Competitive Adaptive Reweighted Sampling (CARS), Bootstrap Soft Shrinkage (BOSS), and Uninformative Variable Elimination (UVE) algorithms. Finally, Support Vector Machine (SVM), Convolutional Neural Network (CNN), and Back Propagation Neural Network (BP) models were developed for rapid detection of crab origin. The results demonstrated that MSC provided the best preprocessing performance for NIR spectral data from both the abdomen and back of the crabs. For abdomen data, the SVM model developed using feature wavelengths selected by the CARS algorithm after MSC preprocessing achieved the highest accuracy (*Acc*) of 90.00%, with precision (*P*), recall (*R*), and F1-score for crabs from Weishan Lake at 89.29%, 86.21%, and 87.72%, respectively; for crabs from Tuo Lake at 86.96%, 95.24%, and 90.91%; and for crabs from Hongze Lake at 90.00%, 93.10%, and 91.53%. For carapace data, the SVM model based on wavelengths selected by the BOSS algorithm after MSC pretreatment achieved the best performance, with an *Acc* of 87.50%, and *P*, *R*, and *F*1 for crabs from Weishan Lake at 77.14%, 93.10%, and 84.38%; for Tuo Lake crabs at 100%, 90.47%, and 95.00%; and for Hongze Lake crabs at 92.31%, 80.00%, and 85.71%. In conclusion, NIR spectroscopy can effectively detect the origin of Chinese mitten crabs, providing technical support for developing rapid detection instruments and thereby safeguarding consumer rights.

## 1. Introduction

The Chinese mitten crab, also known as the hairy crab, is rich in essential nutrients such as fatty acids, proteins, vitamins, and trace elements. It is also a crucial raw material for producing various food products, including crab roe sauce, crab powder, and marinated crabs [1]. Significant differences exist in the composition of fatty acids, flavor compounds, and mineral elements among hairy crabs from different regions. Fatty acids such as ω-3 and ω-6 can reduce the risk of cardiovascular diseases, while flavor compounds like amino acids, nucleotides, and organic acids directly impact the taste. Mineral elements such as calcium, phosphorus, potassium, and magnesium are also vital for human health [2,3]. These variations in taste and nutritional composition among crabs from different origins lead to substantial price differences in the market. Currently, the market is inundated with counterfeit crabs, with crabs from other regions often mislabeled as premium brands like ‘Yangcheng Lake’ and ‘Gucheng Lake’, resulting in inflated prices. For example, although the annual production of Yangcheng Lake hairy crabs is approximately 1500 tons, the market supply exceeds 10,000 tons [4]. This highlights the urgent need for geographical origin identification of hairy crabs, as fraudulent practices have significantly harmed consumer rights. Due to the difficulty in distinguishing crabs from different regions through sensory evaluation alone, identification largely depends on physicochemical methods, which lack the speed required for effective market regulation. Therefore, developing rapid detection techniques for hairy crab origin identification is crucial for regulating the market and protecting consumers’ rights.

To address the issue of geographical origin identification of hairy crabs, researchers have explored various detection methods, including sensory evaluation, stable isotope analysis, mineral element fingerprinting, and chromatographic techniques. Sensory evaluation primarily relies on the aroma and morphological characteristics of the crabs. For example, Lu et al. found that the volatile compounds in the hepatopancreas of hairy crabs vary by region, enabling identification through sensory perception [5]. Similarly, Xu et al. demonstrated that the origin of hairy crabs can be determined by manually examining the carapace, frontal teeth, lateral teeth, and leg characteristics [4]. Regarding stable isotope analysis, Luo et al. utilized strontium (Sr) isotopic analysis to measure the 87Sr/86Sr ratio in the muscle and shell of hairy crabs, achieving precise origin identification with an accuracy of up to 99.4% [2]. For mineral element fingerprinting, Xue et al. conducted biochemical analyses to assess the content and proportions of specific mineral elements within the crabs, discovering that the mineral element fingerprint stabilizes after 3~4 months of cultivation, facilitating reliable origin traceability [6]. Chromatographic methods are also effective for identifying the origin and internal composition of crabs. Yao et al. employed high-performance liquid chromatography (HPLC) to quantify seven amino acids—Cys, Gly, Arg, Val, Met, Pro, and Tyr—in the muscle of crabs from different regions, which allowed for origin and species identification [7]. Wang et al., utilized gas chromatography (GC) to analyze the content of fatty acids, including EPA and DHA, in crab muscle tissue, enabling traceability of crab origin and flavor components [8].

Although the aforementioned methods can accurately identify the geographical origin of the Chinese mitten crab, the sensory-based odor evaluation is highly subjective and heavily reliant on human expertise, and morphological analysis is cumbersome. Stable isotope analysis, mineral element fingerprinting, and chromatographic techniques all face challenges related to operational complexity, slow detection speed, and the need for destructive testing of the crabs. These limitations make it difficult to address the chaotic state of the Chinese mitten crab market, highlighting the urgent need for a rapid and non-destructive method for origin identification. NIR spectroscopy offers several advantages, including rapid, non-destructive testing without the need for sample preparation, and it has seen numerous successful applications in the quality assessment of crabs [9]. For instance, Wold et al. utilized NIR spectroscopy to measure the edible meat content in king crab legs, achieving a coefficient of determination of 0.85, which demonstrates NIR’s capability to penetrate the crab shell and assess internal components. NIR spectroscopy has also been successfully applied in geographical origin traceability for agricultural products and food items [10]. For example, Li et al., achieved non-destructive identification of duck egg origin using visible-NIR spectroscopy with an accuracy of 100.00% [11]. Additionally, Arndt et al. employed NIR to distinguish the geographical origin of Prunus dulcis MILL [12]. Similar applications have been reported for the traceability of yams, tea leaves, and other agricultural products [13,14]. Considering the compositional differences among Chinese mitten crabs from various regions, variations in the number and distribution of hydrogen-containing groups, such as C-H, O-H, and N-H, in their internal components can be expected. The successful applications of NIR spectroscopy in origin identification of agricultural products and food, as well as in crab quality assessment, suggest that NIR can capture these differences. Therefore, NIR spectroscopy offers a promising theoretical approach for the geographical origin identification of the Chinese mitten crab.

This study builds upon previous research on the geographical origin identification of Chinese mitten crabs and the application of NIR spectroscopy in tracing the origin of agricultural products and food. This study aims to develop a rapid detection method for the geographical origin of Chinese mitten crabs using NIR spectroscopy by incorporating effective methodologies from prior work. The approach involves collecting spectral data from the abdomen and carapace of crabs from different origins, applying spectral preprocessing algorithms to clean the data, and selecting key spectral features indicative of origin through feature wavelength algorithms. Finally, machine (deep) learning models are employed to construct a rapid detection model for crab origin identification. Compared to existing crab origin identification methods, the main contributions of this study include: (1) proposing a rapid detection method for the geographical origin of Chinese mitten crabs using NIR spectroscopy; (2) identifying that the feature wavelengths of Chinese mitten crabs from different regions are primarily concentrated between 10,000 cm^−1^ and 9500 cm^−1^, and between 9000 cm^−1^ and 8500 cm^−1^; and (3) demonstrating that NIR data collected from the ventral side of crabs provides more accurate origin identification.

## 2. Methods and Materials

### 2.1. Sample Preparation

**Figure 1:**
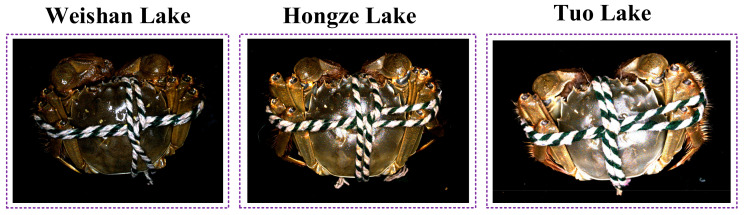
Crabs from three different regions.

This study selected 390 Chinese mitten crabs from three different regions as experimental materials. The crabs were purchased during their sexual maturation season in October 2023 from Weishan Lake, Shandong Province; Tuo Lake, Anhui Province; and Hongze Lake, Jiangsu Province, with 130 crabs from each location. All crabs were of the same species, the Chinese mitten crab, and these regions are recognized as key production areas in the Chinese crab market. To minimize the influence of sex on the origin information of the crabs, the male-to-female ratio was controlled at 1:1 across all groups. Each crab weighed between 100 and 150 g, and all crabs were fresh and alive. Figure 1 shows the crabs from the three regions. The external appearances of the crabs made it difficult to distinguish their origins, as variations were mainly due to individual differences among crabs.

### 2.2. The NIR Spectral Data Collection System for Crabs

**Figure 2:**
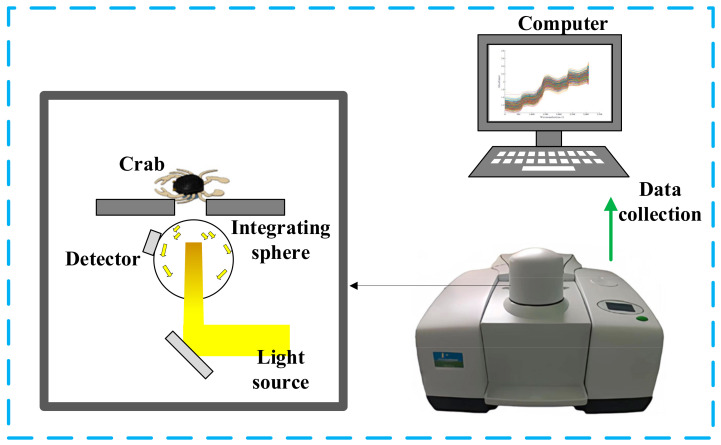
The NIRS data collection system for crab.

The NIR diffuse reflectance spectroscopy data acquisition system for crabs is shown in Figure 2. This system mainly consists of a Fourier transform near-infrared (FT-NIR) spectrometer and a computer. The FT-NIR spectrometer, produced by PerkinElmer (Spectrum Two), features a core diffuse reflectance data acquisition component composed of a light source, an integrating sphere, and a detector. The NIR spectral data acquisition range is from 10,000 to 3800 cm^−1^, with a spectral resolution of 2 cm^−1^. Light emitted from the source is uniformly distributed through the integrating sphere and projected onto the surface of the crab sample. The crabs absorb specific wavelengths of light, leading to diffuse reflection. The reflected spectral information is captured by the detector, acquiring the diffuse reflectance data of the crabs. The computer is connected to the FT-NIR spectrometer and is used to set acquisition parameters and store spectral data. In this study, the binding ropes were removed from the 390 crabs from the three origins. Excess moisture on the crab surface was then wiped off using quick-absorption paper. NIR diffuse reflectance spectra of the abdomen and carapace of each crab were collected separately. For each crab, spectral data from both sides were collected three times, and the average of these measurements represented the NIR spectral data of the sample. In total, 780 NIR spectral datasets were obtained, with each crab contributing two spectra corresponding to its dorsal and ventral sides.

### 2.3. Dataset Preparation

The study collected spectral data from 390 crabs. These crabs were divided into a training set and a test set before developing a crab origin classification model using machine (deep) learning algorithms. The proportion and method of splitting the spectral data significantly impact the performance of the origin classification model. Insufficient NIR spectral data in the training set can result in an underfitted model with inadequate predictive capability for the test set and new crab samples. Conversely, having an excessive number of training samples that lack representativeness can lead to model overfitting. Therefore, a reasonable division of the training and test sets is crucial for developing a robust origin prediction model. This study utilized the SPXY (Sample Set Partitioning Based on Joint X-Y Distance) algorithm to divide the crab dataset. This algorithm evaluates sample similarity based on the joint X-Y distance between NIR spectral samples of crabs. As a result, the 390 crab samples were divided into a training set of 310 samples and a test set of 80 samples, ensuring representativeness [15]. Table 1 shows the distribution of crab samples from the three origins in the training and test sets.

### 2.4. Pretreatment of Spectral Data

During the acquisition of NIR diffuse reflectance spectra of crabs, factors such as fluctuations in experimental temperature and the rough surfaces of crab shells can introduce noise into the spectral data. This noise can interfere with the extraction of characteristic wavelengths related to crab origin and hinder the development of a robust crab origin detection model. Therefore, noise reduction is essential prior to processing the NIR spectral data of crabs to ensure the accurate extraction of characteristic wavelengths related to crab origin. This study employed three commonly used spectral data preprocessing algorithms: Multiplicative Scatter Correction (MSC), Standard Normal Variate (SNV), and Savitzky–Golay (SG) smoothing. The MSC algorithm improves the accuracy of spectral data by correcting for multiple scatterings [16]; the SNV algorithm eliminates variations in the physical properties of samples through standardization [17]; and the SG convolution smoothing algorithm reduces noise while preserving key features by applying local polynomial regression smoothing [18]. MSC and SNV effectively remove scattering noise caused by crab shells, while SG smoothing efficiently reduces high-frequency noise in NIR spectral data arising from instruments, environmental factors, and sample characteristics.

### 2.5. Feature Selection for Spectral Data

The preprocessed NIR spectral data of crabs still contain 3101 dimensions. These high-dimensional data include many wavelength points that are unrelated to the origin information of crabs. Directly modeling with 3101-dimensional data would significantly reduce the detection speed of the model and increase the risk of overfitting due to the inclusion of excessive redundant information as features. Therefore, it is crucial to select characteristic wavelength points that reflect the origin information of crabs from the 3101-dimensional data. In this study, BOSS, CARS, and UVE algorithms were used to extract representative characteristic wavelengths for crab origin detection. By comparing the accuracy of the models, the optimal feature wavelength selection algorithm for determining the origin of crabs was identified.

The BOSS algorithm is a variable selection method used for selecting feature wavelengths [19]. It combines the Model Population Analysis (MPA), a soft shrinkage strategy, and Weighted Bootstrap Sampling (WBS) to extract information from the regression coefficients of PLS (Partial Least Squares) models. In each iteration, the algorithm uses the absolute values of regression coefficients as variable weights and generates sub-models using weighted bootstrap sampling. Next, Model Population Analysis is used to update the variable weights. This process follows a soft shrinkage rule, meaning that instead of directly eliminating insignificant variables, the algorithm assigns them smaller weights. The algorithm iterates until the number of variables is reduced to one and ultimately selects the optimal variable set with the lowest Root Mean Square Error of Cross-Validation (RMSECV).

The UVE algorithm is based on the PLS model and works by adding an equal number of random noise variables to the original spectral data [20]. The algorithm then performs PLS regression analysis on this combined data. During multiple cross-validations, the stability of each wavelength variable’s regression coefficient is calculated as the ratio of its mean to its standard deviation. By comparing these ratios, the UVE algorithm eliminates wavelength variables that exhibit statistical characteristics similar to those of the noise variables, indicating that these variables contribute little to the model.

The CARS algorithm uses Monte Carlo sampling and an exponential decay function to adaptively adjust the selection probability of each wavelength band, ultimately identifying the optimal combination of bands that most significantly contribute to the model’s performance [21]. In each iteration, the algorithm evaluates the importance of each wavelength based on the regression coefficients of the PLS model and selects characteristic wavelengths according to the absolute values of these coefficients as weights. Through multiple iterations, the CARS algorithm gradually eliminates less contributive wavelengths, ultimately determining the optimal set of characteristic wavelengths.

### 2.6. Modeling Methods

The NIR spectral data of crabs, even after feature wavelength selection, cannot directly identify the crab origins. Thus, constructing classification models using the extracted spectral features as inputs is essential for origin identification. Classification models can directly predict the origin of crabs. In this study, SVM (Support Vector Machine), CNN, and BP (Backpropagation) neural networks were employed to build non-destructive crab origin detection models. SVM classifies data by finding the optimal hyperplane in the feature space that maximizes the margin between different class data points. Using kernel tricks, SVM effectively handles linearly inseparable data by mapping it to a higher-dimensional space to find the optimal boundary, which is solved through Lagrange multiplier optimization. SVM exhibits strong robustness to non-linear data, making it suitable for crab origin classification tasks [22].

**Figure 3:**
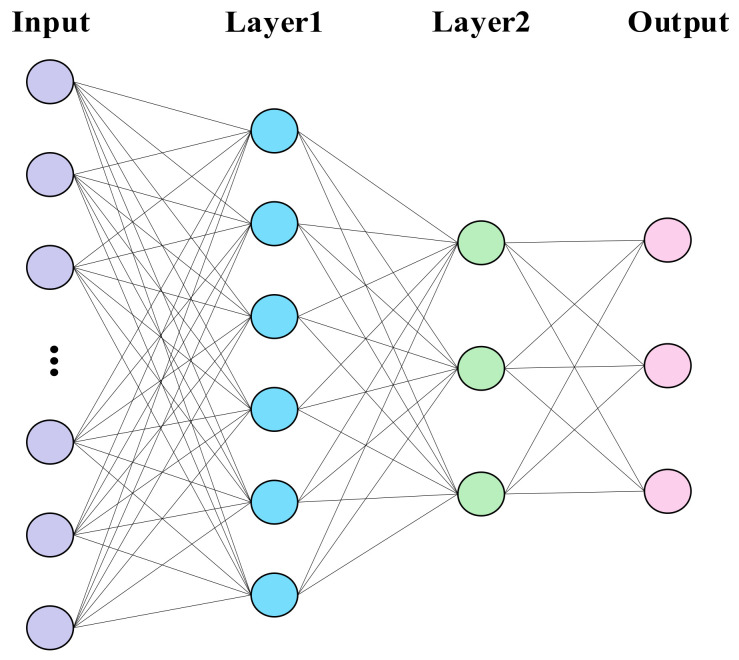
BP neural network for crab origin classification.

The BP neural network is a multilayer feedforward network that learns through forward and backward propagation processes. In forward propagation, input signals are processed by each neuron layer to produce outputs. Backward propagation adjusts the network weights based on output errors and minimizes the loss function through iterative training [23]. The structure of the BP neural network used in this study is shown in Figure 3. The number of neurons in the input layer corresponds to the number of feature wavelength points selected by the feature extraction algorithms mentioned above. Layer 1 and Layer 2 contain six and three neurons, respectively, while the output layer has three neurons representing the three crab origins.

**Figure 4:**
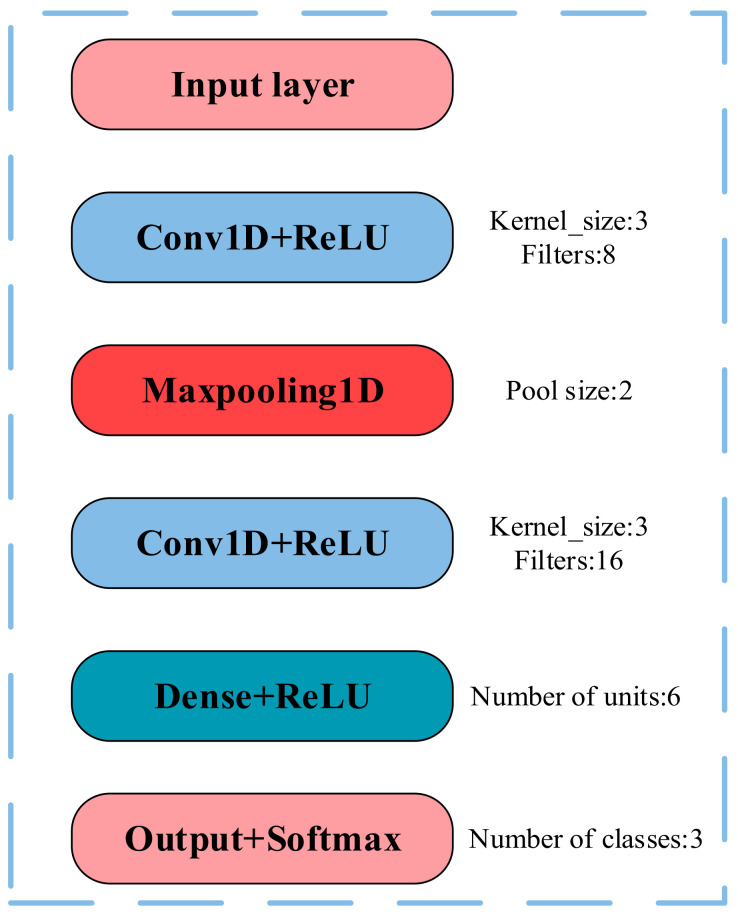
CNN for crab origin classification.

CNN utilizes local connections and weight sharing, granting it powerful information processing capabilities in classification tasks, and it has been widely applied in the field of spectral data classification [24]. In this study, a six-layer one-dimensional spectral convolutional neural network (1D-CNN) was constructed using the feature wavelengths extracted by the aforementioned algorithms as inputs, as shown in Figure 4. The network consists of one input layer, two 1D convolution layers (Conv1D), one 1D max-pooling layer (MaxPooling1D), one fully connected layer (Dense), and one output layer. The two Conv1D layers have kernel sizes of 3, with 8 and 16 kernels, respectively, and are primarily used to automatically extract features related to the crab origins [25]. The MaxPooling1D layer has a pool size of 2, which reduces the dimensionality of the crab spectral features and accelerates the model’s convergence. The Dense layer consists of six neurons, while the output layer provides information on the origins of crabs from three different locations. The outputs of the Conv1D and Dense layers are activated using the ReLU function, enhancing the non-linear expression capability of the CNN [26]. The output layer uses the softmax function, allowing the model to output the probabilities of the crabs belonging to each of the three origins.

### 2.7. Indicators for Model Evaluation

Common evaluation metrics for classification models include accuracy (*Acc*), recall (*R*), precision (*P*), and F1 score (*F*1). Accuracy reflects the overall classification performance of the model in determining crab origin, while recall, precision, and F1 score indicate the model’s discriminative performance for crabs from each origin [27]. The calculation formulas for these metrics are presented in Equations (1)~(4).

(1) $$Acc=\frac{TP+TN}{TP+TN+FP+FN}$$

(2) $$P=\frac{TP}{TP+FP}$$

(3) $$R=\frac{TP}{TP+FN}$$

(4) $$F1=\frac{2\times P\times R}{P+R}$$

In these equations, *TP* (True Positive) represents the number of crab samples that are actually positive and correctly predicted as positive; *FN* (False Negative) refers to the number of crab samples that are actually positive but predicted as negative. *TN* (True Negative) indicates the number of samples that are actually negative and correctly predicted as negative, while *FP* (False Positive) represents the number of samples that are actually negative but predicted as positive. In this study, when calculating the *Acc*, *P*, *R*, and *F*1 for Weishan Lake, the crab samples from Weishan Lake are treated as positive samples, while samples from Tuo Lake and Hongze Lake are considered negative samples. The calculation method for crabs from other origins follows a similar approach.

## 3. Results and Discussion

### 3.1. Analysis of Crab Abdomen and Carapace Spectral Data from Different Origins

**Figure 5:**
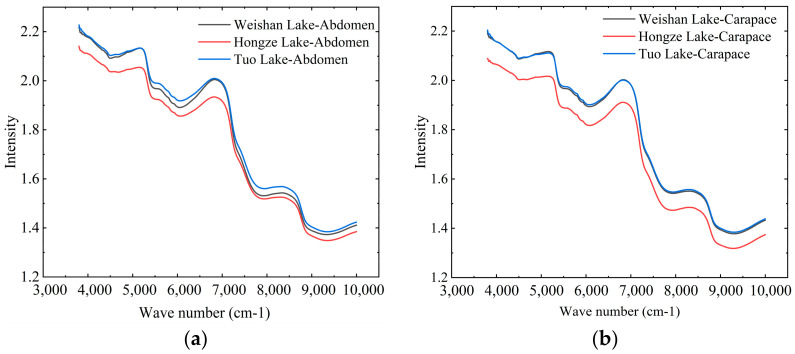
Raw spectral data of crab abdomen and carapace. (**a**) Crab abdomen. (**b**) Crab carapace.

The mean values of the abdomen and carapace raw spectral data of crabs from Weishan Lake, Hongze Lake, and Tuo Lake are shown in Figure 5a,b. It is evident that the peak and trough distributions of the abdomen and carapace spectra are consistent across crabs from all three origins. In the abdomen data, distinct peaks appear near 5100 cm^−1^, 7000 cm^−1^, and 8500 cm^−1^ for crabs from all three locations. However, the mean spectral intensity of Hongze Lake crabs shows significant differences compared to those from Weishan Lake and Tuo Lake, which also display notable differences in the intensity range of 5400~6600 cm^−1^ and 7600~8600 cm^−1^. For the carapace data, prominent peaks are observed around 5200 cm^−1^, 6900 cm^−1^, and 8400 cm^−1^. Again, the mean spectral intensity of Hongze Lake crabs differs significantly from those of the other two locations, while the mean intensity values for Tuo and Weishan Lake crabs are comparatively lower. These differences in mean spectral intensity highlight the capability of NIR diffuse reflectance spectroscopy to capture distinctions among crabs from different origins.

### 3.2. Comparison of Different Spectral Pretreatment Methods

To compare the preprocessing effects of the SG, MSC, and SNV algorithms on crab NIR spectral data and determine the optimal pretreatment method, this study applied these algorithms to preprocess the full-spectrum data of crabs and then established origin prediction models using the SVM model. The prediction results for the test set crab samples are shown in Table 2. For abdomen data, the MSC algorithm yielded the best performance, achieving an overall *Acc* of 80.00%. Specifically, the *P*, *R*, and *F*1 for Tuo Lake crabs were 65.22%, 71.43%, and 68.18%, respectively; for Weishan Lake crabs, *P*, *R*, and *F*1 were 96.30%, 89.66%, and 92.86%, respectively; and for Hongze Lake crabs, *P*, *R*, and *F*1 were all 79.31%. For carapace spectral data, MSC pretreatment also delivered the best results, with an *Acc* of 71.60%. The *P*, *R*, and *F*1 for Tuo Lake crabs were 61.54%, 76.19%, and 68.08%, respectively; for Weishan Lake crabs, *P*, *R*, and *F*1 were all 65.52%; and for Hongze Lake crabs, *P*, *R*, and *F*1 were 88.00%, 73.33%, and 80.00%, respectively. Additionally, the predictive performance of the abdomen NIR spectral data surpassed that of the carapace NIR spectral data. The prediction performance of both abdomen and carapace NIR spectral data, when pretreated with SG, MSC, or SNV algorithms, was superior to that of the raw spectral data. This may be related to the complex surface structure of crabs, which has certain irregularities and glossiness, leading to significant scattering effects and baseline drift in the spectral data. MSC is more effective in correcting the spectral deviations caused by surface scattering, particularly in baseline correction. Although SNV can also reduce scattering effects, it is relatively limited in dealing with baseline drift; SG is mainly used for spectral smoothing and differentiation, which may result in the loss of some useful information. Therefore, the MSC-pretreated NIR spectral data of both the abdomen and carapace of the crabs were selected for subsequent analysis in this study.

### 3.3. Sensitive Band Analysis of Crab

**Figure 6:**
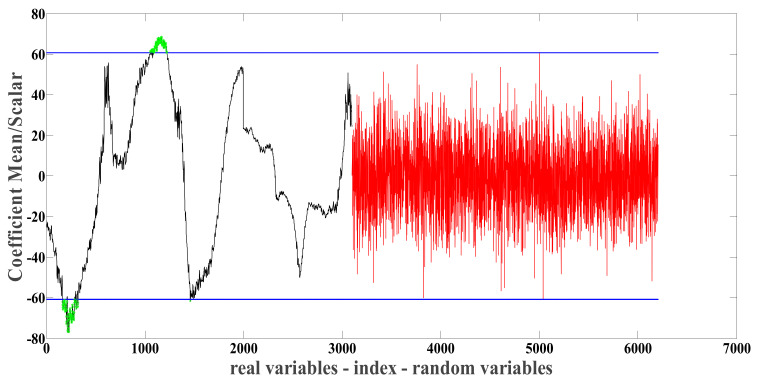
Feature wavelength point selection using UVE (Uninformative Variable Elimination).

After MSC preprocessing, the NIR spectral data from the abdomen and carapace of the crabs were subjected to feature wavelength selection using the UVE, the BOSS, and the CARS algorithms to identify key wavelength sets that reflect the crabs’ origin. The results of feature wavelength selection using the UVE algorithm for the abdomen NIR spectral data are shown in Figure 6. The two horizontal blue lines represent the selection thresholds for the variables, with the area inside the blue lines containing useless information and the area outside containing useful information.The black curve represents the stability values of the crab spectral variables, the red curve indicates the stability values of noise variables, and the green points represent the selected spectral feature variables. Ultimately, UVE selected 287 feature wavelengths, with 136 located in the range of 9830 cm^−1^ to 9677 cm^−1^, 149 in the range of 8943 cm^−1^ to 8794 cm^−1^, and 2 in the range of 8539 cm^−1^ to 8538 cm^−1^. By analyzing the absorption characteristics of these bands, we found that these selected feature bands are primarily related to the key chemical components of crabs. Specifically, the band from 9830 cm^−1^ to 9677 cm^−1^ is closely associated with the O-H stretching vibration of water, reflecting variations in the water content of the crabs; the band from 8943 cm^−1^ to 8794 cm^−1^ is related to the C-H stretching vibration of fats, indicating differences in fat content among crabs from different origins; and the band from 8539 cm^−1^ to 8538 cm^−1^ may be associated with specific functional groups in proteins (such as N-H bonds) [28]. The UVE selection process for the NIR spectral data of the carapace of the crabs followed a similar approach.

**Figure 7:**
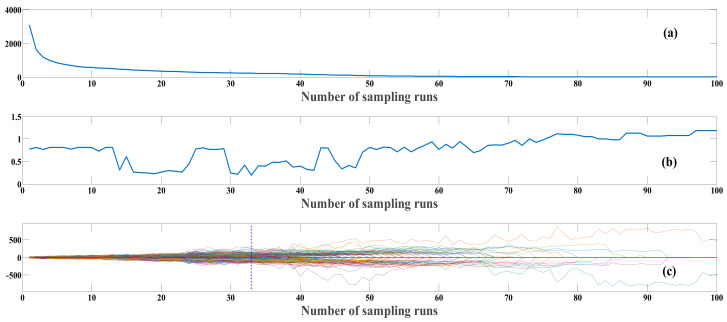
Feature wavelength point selection using CARS (Competitive Adaptive Reweighted Sampling). (**a**) Number of sampled variables; (**b**) RMSECV; (**c**) Regression coefficients path.

The feature wavelength selection process for the crab abdomen data using the CARS algorithm is shown in Figure 7. The Monte Carlo sampling number was set to 100, with five-fold cross-validation. As shown in Figure 7a, the number of selected spectral features decreased as the sampling iterations increased. In Figure 7b, the root mean square error of cross-validation (RMSECV) decreased dynamically from 0 to 33 sampling iterations, eliminating a substantial amount of redundant information unrelated to crab origin. Beyond 33 iterations, the RMSECV began to increase, indicating the removal of useful information. The regression coefficients of each variable at the minimum RMSECV value are indicated by vertical lines in Figure 7c. At this point, 220 feature variables were selected, distributed as follows: 36 feature wavelengths between 9988 cm^−1^ and 9703 cm^−1^, 28 between 9694 cm^−1^ and 9511 cm^−1^, 51 between 9579 cm^−1^ and 9243 cm^−1^, 34 between 9180 cm^−1^ and 9031 cm^−1^, and 71 between 8975 cm^−1^ and 8044 cm^−1^. The spectral range from 9988 cm^−1^ to 9703 cm^−1^ is associated with the O-H stretching vibration of water, indicating its high sensitivity to water content. The ranges from 9694 cm^−1^ to 9511 cm^−1^ and from 9579 cm^−1^ to 9243 cm^−1^ are related to the C-H stretching vibration of fats, reflecting differences in fat content. The range from 9180 cm^−1^ to 9031 cm^−1^ corresponds to the N-H vibration in proteins, while the range from 8975 cm^−1^ to 8044 cm^−1^ may be related to characteristic absorptions of various organic compounds, such as proteins and carbohydrates [29]. The feature wavelength extraction process for the crab carapace spectral data followed a similar approach.

**Figure 8:**
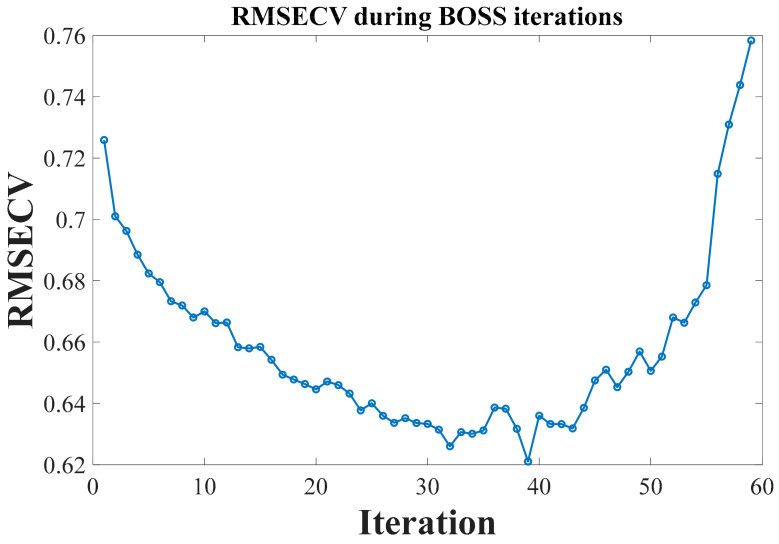
The variation of RMSECV with the number of iterations.

**Figure 9:**
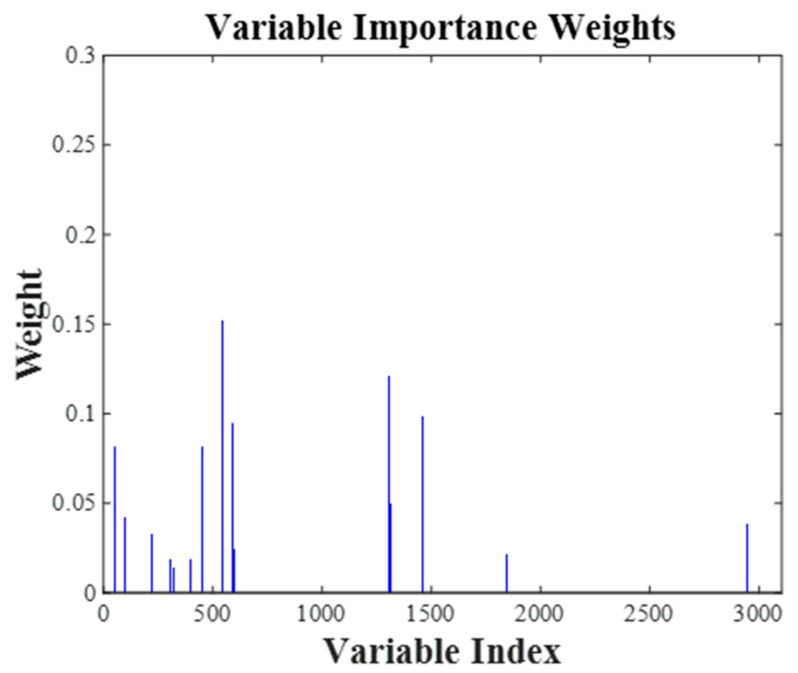
The weight distribution at the 33rd iteration.

**Figure 10:**
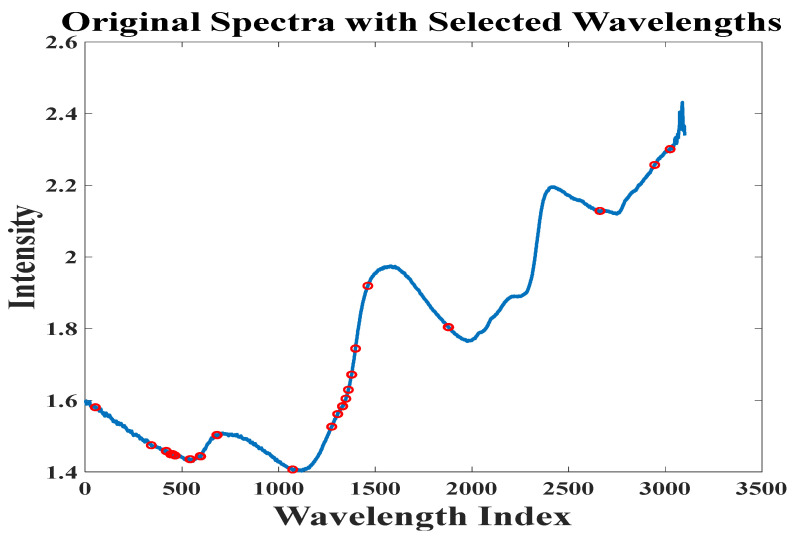
Feature wavelengths selected by BOSS (Bootstrap Soft Shrinkage).

Similarly, the BOSS algorithm was employed to select feature wavelengths that reflect the provenance information of crabs, following a soft-thresholding rule for eliminating irrelevant data. Instead of outright removing wavelengths that do not directly convey provenance information, these points were assigned minimal weights. This iterative process continued until the number of features was reduced to one. In this study, the bootstrap sample count was set to 1000, and the optimal iteration was determined based on the RMSECV at each step, as shown in Figure 8. It was observed that the RMSECV decreased rapidly in the first 39 iterations, then increased sharply from the 39th to the 60th iteration before stabilizing. Thus, the feature set from the 39th iteration was analyzed in detail, with weights assigned to these variables as shown in Figure 9. The BOSS algorithm ultimately selected 29 key variables to reflect the provenance information of the crabs, as depicted in Figure 10, with a generally uniform distribution between 10,000 cm^−1^ and 4000 cm^−1^. This selection is likely related to the overtone absorption of N-H and O-H bonds in crabs. The feature wavelength extraction process for the crab carapace spectral data followed a similar approach.

### 3.4. Analysis of Modeling Results for Crab Abdomen and Carapace

After selecting the feature wavelengths, this study utilized SVM, BP, and CNN to construct origin discrimination models based on the NIR spectral data from the abdomen and carapace of crabs, with the results shown in Table 3 and Table 4. Table 3 presents the models constructed using the abdomen NIR spectral data. For the SVM model, the model using feature wavelengths extracted by the CARS algorithm yielded the best performance, with an overall *Acc* of 90.00%. Specifically, for Weishan Lake crabs, the *P*, *R*, and *F*1 were 89.29%, 86.21%, and 87.72%, respectively. For Tuo Lake crabs, the *P*, *R*, and *F*1 were 86.96%, 95.24%, and 90.91%, respectively. For Hongze Lake crabs, the *P*, *R*, and *F*1 were 90.00%, 93.10%, and 91.53%, respectively. Notably, the model exhibited the best performance in distinguishing Hongze Lake crabs, consistent with the significant differences observed in the abdominal raw NIR spectral data of Hongze Lake crabs compared to those from the other two regions. For the BP model, the feature wavelengths extracted by the BOSS algorithm yielded the best performance, with an *Acc* of 85.00%. In the case of the CNN model, the feature wavelengths extracted by the UVE algorithm led to the highest performance, with an *Acc* of 86.25%.

Table 4 presents the models constructed using carapace NIR spectral data for crab origin discrimination. For SVM, the model with feature wavelengths extracted by the BOSS algorithm achieved the best performance, with an overall *Acc* of 87.50%. Specifically, for Weishan Lake crabs, the *P*, *R*, and *F1* were 77.14%, 93.10%, and 84.38%, respectively; for Tuo Lake crabs, the *P*, *R*, and *F*1 were 100%, 90.47%, and 95.00%, respectively; and for Hongze Lake crabs, the *P*, *R*, and *F*1 were 92.31%, 80.00%, and 85.71%, respectively. For the BP model, the feature wavelengths extracted by the BOSS algorithm also yielded the best performance, with an *Acc* of 77.50%. For the CNN model, the CARS algorithm provided the optimal feature wavelengths, resulting in an *Acc* of 77.50%. Overall, although both carapace and abdomen NIR spectral data enabled the discrimination of crab origins, the models based on abdomen NIR spectral data consistently outperformed those based on carapace data. This suggests that collecting NIR diffuse reflectance spectra from the crab’s abdomen is more suitable for origin discrimination of crabs.

The results of this study indicate that NIR spectroscopy can also be used to differentiate the origins of Chinese mitten crabs, showing consistency with sensory analysis, stable isotope analysis, mineral element fingerprinting, and chromatography. Compared to the sensory identification methods used by [4,5] our approach does not rely on the examiner’s expertise and is faster. Additionally, compared to [2], who analyzed the 87Sr/86Sr ratio in crab meat and shells using strontium isotopes, and [6], who assessed specific mineral element content and ratios within crabs, or [7], who utilized high-performance liquid chromatography to measure amino acid content in crabs from various regions, their accuracy exceeded 99%. Although the detection accuracy of our study is slightly lower, the method used here is simpler, faster, and more suitable for real-time crab origin detection. Therefore, this study aligns more closely with the market demand for rapid origin detection of crabs, directly addressing the problem of crab origin identification.

However, this study only used Chinese mitten crabs from three specific regions as experimental materials and did not attempt to analyze crabs from other origins. But the method used in this study can also be extended to identify the origins of crabs from other regions. In future research, we plan to collect NIR spectral data from crabs of additional regions using the spectral acquisition method developed in this study. We will then use the feature wavelength extraction and modeling methods presented here to update and optimize the model, enhancing its adaptability to crabs from various regions. Furthermore, we plan to develop a corresponding fiber optic spectrometer and portable detection equipment based on the sensitive wavelengths identified for crab origin. This aims to reduce the equipment cost of NIR spectroscopy and enhance detection speed, ultimately addressing the issue of unclear crab origin in the market.

### 3.5. Optimization of the Optimal Model Parameters

The above results indicate that for the SVM model, the crab origin classification model based on feature wavelengths extracted by the CARS algorithm performs best; for the BP model, the model based on feature wavelengths extracted by the BOSS algorithm performs best; and for the CNN model, the model based on feature wavelengths extracted by the CARS algorithm performs best. To determine the optimal parameters for the models in crab origin detection, this study used grid search for parameter optimization. The core principle is to exhaustively search all possible combinations of hyperparameters to find the optimal model configuration. For the SVM model, the kernel function, penalty parameter (C), and kernel coefficient (gamma) are the important hyperparameters affecting the performance of the crab origin classification model. For the BP model, the number of training iterations, learning rate, and stopping error are the key hyperparameters affecting its performance. Similarly, for the CNN model, the initial learning rate, mini-batch size, and maximum number of training epochs are the important hyperparameters. The optimal model parameters obtained for each of the three models after grid search are shown in Table 5.

Subsequently, we established crab origin classification models using the optimal hyperparameter combinations and compared the results with those obtained without parameter optimization, as shown in Table 6. It can be observed that the performance of the optimized SVM model remained consistent with that before optimization. However, the accuracy (*Acc*) of the BP model increased from 85.00% to 86.25%, and the accuracy of the CNN model improved from 86.25% to 87.50%. Nevertheless, the performance was still lower than that of the SVM model constructed using NIR data from the crabs’ abdomen and feature wavelengths extracted by the CARS algorithm. This may be due to the fact that SVM are particularly effective for small to medium-sized datasets, making them suitable for our dataset, which consists of limited samples from three regions. In contrast, CNN and BP models generally require larger datasets to achieve robust generalization capabilities.

### 3.6. Analysis of the Impact of Dataset Size on the Optimal Model

To verify the impact of dataset size on the performance of the optimized models, this study randomly selected 100 and 80 crab samples from each region, forming two smaller datasets with sizes of 300 (dataset-300) and 240 (dataset-240) samples, respectively. The crab abdomen data were then divided and preprocessed using the same dataset partitioning method and the MSC preprocessing algorithm. The optimal feature wavelength selection and modeling methods were applied to establish crab origin detection models for each dataset size. The modeling results are shown in Table 7.

It can be observed that when the dataset size is 300 samples, compared to the original dataset of 390 samples, the classification performance of the SVM, BP, and CNN models for crab origin decreased, with accuracy rates of 87.50%, 83.93%, and 85.71%, respectively. When the dataset size was reduced to 240 samples, the classification performance of SVM, BP, and CNN further decreased, with accuracy rates of 86.67%, 84.44%, and 84.44%, respectively. This indicates that the larger the dataset size, the better the model’s classification performance. However, even with a smaller dataset, it was demonstrated that NIR spectroscopy can effectively differentiate crab origins. In future research, we will continue to expand the dataset size to further improve the model’s performance.

## 4. Conclusions

This study collected NIR diffuse reflectance spectral data from the abdomen and carapace regions of 390 Chinese mitten crabs. For each crab, three spectral measurements were taken from both the abdomen and the carapace, with the average values representing the spectral data for each individual. The NIR spectral data were pretreated using the MSC algorithm. Subsequently, feature selection was performed using the CARS, the BOSS, and the UVE algorithms. Finally, the SVM, the BP, and the CNN models were developed to detect the geographical origin of the crabs, providing technical support for the rapid detection of crab origin using NIR spectroscopy. The main findings are as follows:

(1) The characteristic wavelengths reflecting the geographical origin of crabs were primarily distributed between 10,000 cm^−1^ to 9500 cm^−1^ and 9000 cm^−1^ to 8500 cm^−1^.
(2) For abdomen spectral data, the SVM model based on the feature wavelengths selected by the CARS algorithm demonstrated the best performance in discriminating crab origin, with an overall *Acc* of 90.00%. Specifically, for crabs from Weishan Lake, *P*, *R*, and *F*1 were 89.29%, 86.21%, and 87.72%, respectively; for crabs from Tuo Lake, these metrics were 86.96%, 95.24%, and 90.91%; and for crabs from Hongze Lake, they were 90.00%, 93.10%, and 91.53%.
(3) For carapace spectral data, the SVM model based on the feature wavelengths selected by the BOSS algorithm exhibited the highest discriminative performance, with an accuracy of 87.50%. For Weishan Lake crabs, *P*, *R*, and *F*1 were 77.14%, 93.10%, and 84.38%, respectively; for Tuo Lake crabs, the values were 100%, 90.47%, and 95.00%; and for Hongze Lake crabs, the corresponding metrics were 92.31%, 80.00%, and 85.71%.

These results indicate that MSC pretreated combined with feature selection and SVM modeling shows substantial potential for the identification of crab origin, with particularly enhanced effectiveness when utilizing abdomen spectral data.

## Figures and Tables

**Table 1:** The detailed distribution of the crab.

	Weishan Lake	Tuo Lake	Hongze Lake	Total
Training set	101	109	100	310
Test set	29	21	30	80
Total	130	130	130	390

**Table 2:** Results of SVM based on different pretreatment methods.

Pretreatment	VN	Results	Abdomen					Carapace			
		Category	Number	*P*	*R*	*F*1	*Acc*	*P*	*R*	*F*1	*Acc*
None	3101	WL	29	50.00%	68.97%	57.97%	58.75%	80.95%	58.62%	68.00%	56.20%
		TL	21	60.00%	42.86%	50.00%		48.57%	80.95%	78.63%	
		HL	30	72.00%	60.00%	65.45%		45.83%	36.67%	40.74%	
MSC	3101	WL	29	96.30%	89.66%	92.86%	80.00%	65.52%	65.52%	65.52%	71.60%
		TL	21	65.22%	71.43%	68.18%		61.54%	76.19%	68.08%	
		HL	30	79.31%	79.31%	79.31%		88.00%	73.33%	80.00%	
SG	3101	WL	29	77.78%	72.41%	75.00%	67.50%	52.38%	75.86%	61.97%	63.70%
		TL	21	46.43%	61.90%	53.06%		38.10%	57.14%	45.72%	
		HL	30	76.92%	66.67%	71.43%		87.50%	61.54%	72.26%	
SNV	3101	WL	29	86.67%	89.66%	88.14%	76.20%	64.29%	62.07%	63.16%	70.00%
		TL	21	56.52%	61.90%	59.09%		61.54%	76.19%	68.07%	
		HL	30	81.48%	73.33%	77.19%		84.62%	73.33%	78.58%	

VN: Number of Variables. WL: crab from Weishan Lake. TL: crab from Tuo Lake. HL: crab from Hongze Lake. MSC: Multiplicative Scatter Correction. SG: Savitzky–Golay Smoothing. SNV: Standard Normal Variate. *P*: Precision. *R*: Recall. *Acc*: Accuracy. *F*1: F1 Score.

**Table 3:** Modeling results for crab abdomen data.

Model	Processing	VN	*Acc*%	Results				
				Category	Number	*P*%	*R*%	*F*1%
SVM	CARS	220	90.00	WL	29	89.29	86.21	87.72
				TL	21	86.96	95.24	90.91
				HL	30	90.00	93.10	91.53
	UVE	287	81.25	WL	29	78.13	86.21	81.97
				TL	21	84.21	76.19	80.00
				HL	30	80.00	82.76	81.36
	BOSS	29	87.5	WL	29	86.21	86.21	86.21
				TL	21	90.00	85.71	87.81
				HL	30	90.00	93.10	91.52
BP	CARS	220	82.50	WL	29	77.42	82.76	80.00
				TL	21	90.00	85.71	87.80
				HL	30	80.00	82.76	81.36
	UVE	287	75.00	WL	29	73.33	75.86	74.58
				TL	21	72.00	85.71	78.26
				HL	30	80.00	66.67	72.73
	BOSS	29	85.00	WL	29	89.29	86.21	87.72
				TL	21	79.17	90.48	84.44
				HL	30	85.71	80.00	82.76
CNN	CARS	220	83.75	WL	29	88.46	79.31	83.64
				TL	21	79.17	90.48	84.44
				HL	30	83.33	83.33	83.33
	UVE	287	86.25	WL	29	92.59	86.21	89.29
				TL	21	80.00	95.24	86.96
				HL	30	85.71	80.00	82.76
	BOSS	29	77.50	WL	29	76.67	79.31	77.96
				TL	21	80.95	80.95	80.95
				HL	30	75.86	73.33	74.58

VN: Number of Variables. WL: crab from Weishan Lake. TL: crab from Tuo Lake. HL: crab from Hongze Lake. CARS: Competitive Adaptive Reweighted Sampling. UVE: Uninformative Variable Elimination. BOSS: Bootstrap Soft Shrinkage. *P*: Precision. *R*: Recall. *Acc*: Accuracy. *F*1: F1 Score. SVM: Support Vector Machine. BP: Backpropagation Neural Network. CNN: Convolutional Neural Network.

**Table 4:** Modeling results for crab carapace data.

Model	Processing	VN	*Acc*%	Results				
				Category	Number	*P*%	*R*%	*F*1%
SVM	CARS	194	85.00	WL	29	85.71	82.76	84.21
				TL	21	94.74	85.71	90.00
				HL	30	78.79	86.67	82.54
	UVE	306	70.00	WL	29	70.37	65.52	67.86
				TL	21	77.78	66.67	71.79
				HL	30	65.71	76.67	70.77
	BOSS	26	87.50	WL	29	77.14	93.10	84.38
				TL	21	100	90.47	95.00
				HL	30	92.31	80.00	85.71
BP	CARS	194	77.50	WL	29	75.86	75.86	75.86
				TL	21	77.78	66.67	71.79
				HL	30	81.82	90.00	85.71
	UVE	306	70.00	WL	29	68.00	58.62	62.96
				TL	21	69.23	85.71	76.59
				HL	30	72.41	70.00	71.19
	BOSS	26	77.50	WL	29	74.07	68.97	71.42
				TL	21	83.33	95.24	88.89
				HL	30	75.86	73.33	74.58
CNN	CARS	194	77.50	WL	29	74.19	79.31	76.67
				TL	21	69.23	85.71	76.59
				HL	30	75.86	73.33	74.58
	UVE	306	62.50	WL	29	60.71	58.62	59.65
				TL	21	62.50	71.43	66.67
				HL	30	64.26	60.00	62.08
	BOSS	26	65.00	WL	29	64.29	62.07	63.16
				TL	21	61.54	76.19	68.08
				HL	30	66.67	60.00	63.16

VN: Number of Variables. WL: crab from Weishan Lake. TL: crab from Tuo Lake. HL: crab from Hongze Lake. CARS: Competitive Adaptive Reweighted Sampling. UVE: Uninformative Variable Elimination. BOSS: Bootstrap Soft Shrinkage. *P*: Precision. *R*: Recall. *Acc*: Accuracy. *F*1: F1 Score. SVM: Support Vector Machine. BP: Backpropagation Neural Network. CNN: Convolutional Neural Network.

**Table 5:** The detailed optimal model parameters.

Model	Parameters	Optimal Parameter
SVM	C	1.0
	gamma	0.5
	kernel function	radial basis function (RBF)
BP	training iterations	2500
	learning rate	0.003
	stopping error	5 × 10^−6^
CNN	training epochs	299
	batch size	4
	initial learning rate	0.0001

**Table 6:** Modeling results before and after parameter optimization.

	Model	*Acc*%	Results				
			Category	Number	*P*%	*R*%	*F*1%
Before Optimization	SVM	90.00	WL	29	89.29	86.21	87.72
			TL	21	86.96	95.24	90.91
			HL	30	90.00	93.10	91.53
	BP	85.00	WL	29	89.29	86.21	87.72
			TL	21	79.17	90.48	84.44
			HL	30	85.71	80.00	82.76
	CNN	86.25	WL	29	92.59	86.21	89.29
			TL	21	80.00	95.24	86.96
			HL	30	85.71	80.00	82.76
After Optimization	SVM	90.00	WL	29	89.29	86.21	87.72
			TL	21	86.96	95.24	90.91
			HL	30	90.00	93.10	91.53
	BP	86.25	WL	29	89.29	86.21	87.72
			TL	21	80.00	95.24	86.95
			HL	30	88.89	80.00	84.21
	CNN	87.50	WL	29	86.21	89.29	87.72
			TL	21	84.00	100.00	91.30
			HL	30	88.89	80.00	84.21

WL: crab from Weishan Lake. TL: crab from Tuo Lake. HL: crab from Hongze Lake. *P*: Precision. *R*: Recall. *Acc*: Accuracy. *F*1: F1 Score. SVM: Support Vector Machine. BP: Backpropagation Neural Network. CNN: Convolutional Neural Network.

**Table 7:** Modeling results of dataset-300 and dataset-300.

	Model	*Acc*%	Results				
			Category	Number	*P*%	*R*%	*F*1%
Dataset-300	SVM	87.50	WL	19	93.75	78.95	85.72
			TL	18	89.47	94.44	91.89
			HL	19	80.95	89.47	85.00
	BP	83.93	WL	19	84.21	84.21	84.21
			TL	18	88.89	88.89	88.89
			HL	19	88.89	84.21	86.49
	CNN	85.71	WL	19	84.21	84.21	84.21
			TL	18	89.47	94.44	91.89
			HL	19	83.33	78.95	81.08
Dataset-240	SVM	86.67	WL	15	92.86	86.67	89.66
			TL	13	80.00	92.31	85.72
			HL	17	87.50	82.35	84.85
	BP	84.44	WL	15	77.78	93.33	84.85
			TL	13	100.00	76.92	86.96
			HL	17	82.35	82.35	82.35
	CNN	84.44	WL	15	80.00	80.00	80.00
			TL	13	91.67	84.62	88.00
			HL	17	77.78	82.35	80.00

WL: crab from Weishan Lake. TL: crab from Tuo Lake. HL: crab from Hongze Lake. *P*: Precision. *R*: Recall. *Acc*: Accuracy. *F*1: F1 Score. SVM: Support Vector Machine. BP: Backpropagation Neural Network. CNN: Convolutional Neural Network.

## Data Availability

The original contributions presented in the study are included in the article, further inquiries can be directed to the corresponding author.
